# A Novel Archaeal Lineage in Boiling Hot Springs around Oyasukyo Gorge (Akita, Japan)

**DOI:** 10.1264/jsme2.ME21048

**Published:** 2021-11-25

**Authors:** Katsuhiro Asamatsu, Kai Yoshitake, Makoto Saito, Wipoo Prasitwuttisak, Jun-ichiro Ishibashi, Akihi Tsutsumi, Nurul Asyifah Mustapha, Toshinari Maeda, Katsunori Yanagawa

**Affiliations:** 1 Faculty of Environmental Engineering, The University of Kitakyushu, Kitakyushu 808–0135, Japan; 2 Department of Earth and Planetary Sciences, Faculty of Science, Kyushu University, Fukuoka 819–0395, Japan; 3 Department of Biological Functions Engineering, Graduate School of Life Sciences and Systems Engineering, Kyushu Institute of Technology, Kitakyushu, 808–0196, Japan

**Keywords:** novel archaeal lineage, thermophile, hyperthermophile, boiling hot spring

## Abstract

A novel deep-branching archaeal lineage was discovered at high-temperature hot springs around Oyasukyo Gorge in Akita Prefecture, Japan. Actively boiling hot spring water contained >1×10^4^ microbes mL^–1^. The microbial community composition assessed by analyzing 16S rRNA gene amplicons revealed that the dominant bacterial phyla were *Proteobacteria* and *Aquificae* (>50% of the microbial composition) in samples collected in 2016 and 2019, respectively. Approximately 10% of the reads obtained in both years were not assigned to any taxonomy. The more detailed phylogenetic positions of the unassigned sequences identified using a clone library and phylogenetic tree showed that they formed a clade that was independent, distantly related to known phyla, and had low similarity (<82%) to all other sequences in available databases. The present results suggest that this novel archaeal phylum-level lineage thrives in boiling hot springs in Japan.

Microbial communities, including as-yet uncultivated lineages, have been surveyed using cultivation-independent molecular biological techniques. The 16S rRNA gene sequencing of microbial populations in natural environments has expanded our knowledge on the microbial diversity, phylogenetic classification, distribution, and evolutionary relationships among prokaryotes. Diverse high-temperature habitats above 80°C have also been investigated from the perspective of the ecological and physiological functions of numerous thermophiles and hyperthermophiles (Takai and Horikoshi, 1999; Hetzer *et al.*, 2007; Kvist *et al.*, 2007; Wilson *et al.*, 2008; Dodsworth *et al.*, 2011; Hou *et al.*, 2013; Song *et al.*, 2013; Sugihara *et al.*, 2016). These studies focused on deep- and shallow-sea hydrothermal systems and terrestrial geothermal systems, particularly those in Yellowstone National Park in the United States (Huber *et al.*, 1998; Hugenholtz *et al.*, 1998; Reysenbach *et al.*, 2000; Blank *et al.*, 2002; Spear *et al.*, 2005; Kozubal *et al.*, 2013; Jay *et al.*, 2018). Unique high-temperature terrestrial environments with various chemical compositions in Japan have also provided large reservoirs of diverse microbial populations. These habitats are dominated by *Aquificales* (Yamamoto *et al.*, 1998; Nishihara *et al.*, 2018; Nishiyama *et al.*, 2018), *Sulfolobus* (Takai and Sako, 1999; Kato *et al.*, 2011; Satoh *et al.*, 2013; Nishiyama *et al.*, 2018), *Thermoprotei* (Kato *et al.*, 2011), *Thermodesulfobacteria* (Nishiyama *et al.*, 2018), *Chloroflexi* (Martinez *et al.*, 2019), and methanogenic archaea (Matsushita *et al.*, 2016). Some previously uncharacterized lineages, such as *Aigarchaeota* (Nunoura *et al.*, 2011), ARMAN (Murakami *et al.*, 2012), HWCGIII (Nunoura *et al.*, 2005), OP1 (Takami *et al.*, 2012), OP5 (Mori *et al.*, 2008), *Thaumarchaeota* (Nishizawa *et al.*, 2013), and THSCG (Kato *et al.*, 2019), are also prominent populations in hot spring environments. Although these descriptions of previously unknown thermophilic and hyperthermophilic lineages have led to advances in extreme microbiology in Japan and elsewhere, the importance of archaea in many environments remains unknown (Adam *et al.*, 2017).

The 16S rRNA gene has been increasingly surveyed in terrestrial geothermal systems using high-throughput DNA sequencing (HTS). This has provided a deeper coverage of microbial communities, which may offer important advantages for detecting low-abundance populations in the rare biosphere (Sogin *et al.*, 2006), as well as for the further identification of new taxa. We herein describe the geomicrobiological characterization of boiling hot springs around Oyasukyo Gorge in Akita Prefecture, Japan. To the best of our knowledge, the molecular ecology of the geothermal area around Oyasukyo Gorge remains unknown. The results obtained revealed the distribution of a novel deep-branching lineage of archaea that is not rare, but is rather an archaeal majority.

## Materials and Methods

### Sample collection and processing

**Fig. 1.**
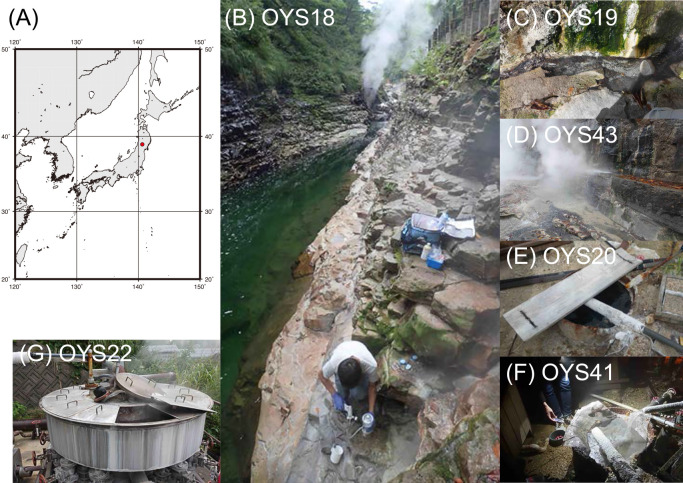
Location and images of sampling sites. (A) Red dot: study sites around Oyasukyo Gorge in northeastern Japan. (B), (C), and (D) Oyasukyo Daifunto. (E) and (F) Oku-Oyasukyo Ooyu hot spring. (G) Storage tank located about midway between Oyasukyo Daifunto and Oku-Oyasukyo Ooyu hot springs.

Hot water samples were collected from two hot springs, Oyasukyo Daifunto (39.01161° N, 140.66079° E, 270‍ ‍m above sea level [a.s.l.]) and Oku-Oyasukyo Ooyu hot spring (38.98962° N, 140.68928° E, 366‍ ‍m a.s.l.), located in the eastern part of Yuzawa city, Akita Prefecture (Fig. 1 and S1). The surrounding region has potential as a location for geothermal power plants (Abe *et al.*, 1979; Naka and Okada, 1992). Oyasukyo is a 60-meter-deep V-shaped gorge created by fluvial erosion of the Minase River. Oyasukyo Daifunto vigorously spouts from cracks in sedimentary rocks at the bottom of a steep cliff. Four hot water samples (OYS18, 19, 41, and 43) were obtained using a sterilized ladle in September 2016 and November 2019. The Oku-Oyasukyo Ooyu hot spring is located ~3‍ ‍km upstream of Oyasukyo. Boiling water blasts from the bottom along the river. The hot spring water samples OYS20 and OYS41 were collected from a shallow well near the flow in 2016 and 2019. The hot water sample, OYS22, was collected in 2016 from a storage tank (39.00159° N, 140.66915° E, 352‍ ‍m a.s.l.), which holds hot spring water pumped from a deep well located approximately midway above the two sites. Table 1 shows the locations, sampling dates, and geochemical characteristics of water samples.

### Water geochemistry

Water temperature, electrical conductivity (EC), oxidation-reduction potential (ORP), and pH were measured on-site using a LAQUA WQ-330J portable water quality meter (Horiba) before sampling. Hot water samples collected using sterile syringes were passed through a 0.45-μm filter. The concentrations of dissolved silica, ammonium ions, and hydrogen sulfide were measured by conventional staining with molybdenum blue (Gieskes *et al.*, 1991), indophenol (Gieskes *et al.*, 1991), and methylene blue (Cline, 1969), respectively. Alkalinity was assessed by potentiometric titration with 0.1 N hydrochloric acid, where the endpoint was calculated by a Gran-function evaluation. Major anions (Cl^–^ and SO_4_^2–^) in the filtrate were analyzed using a Dionex^TM^ X-100 ion chromatograph (Thermo Fisher Scientific). The concentrations of major and minor cations (Na^+^, Mg^2+^, K^+^, and Ca^2+^) and dissolved metal elements (Al and Fe) were measured using a Model 5100 inductively coupled plasma optical emission spectrometer (ICP-OES) (Agilent Technologies). Estimated analytical errors in the chemical ana­lysis were within 5% based on repeated analyses.

### Counting of microbial cells

Portions of water samples were mixed in 3% formaldehyde at room temperature for 2 h, and fixed microbes were collected on polycarbonate Isopore Membrane filters with 0.2-μm pores (Merck KGaA) and then stored at –80°C. Fixed microbes were stained on the filter with 250×SYBR Green I (Thermo Fisher Scientific) in darkness at room temperature for 10‍ ‍min (Yanagawa *et al.*, 2014). The filters were rinsed with TE buffer, mounted on glass slides using VECTASHIELD^®^ mounting medium (H1000; Vector Laboratories), and then covered with glass slips. Cells labeled with green fluorescence were examined using an Eclipse 80i fluorescence microscope (Nikon) equipped with B-2A longpass filter cubes. Cell density was assessed by counting >1×10^3^ cells in at least 25 microscopic fields per filter.

### Microbial 16S rRNA gene abundance

Hot water samples (0.5–3 L) were filtered through a membrane with 0.22-μm pores using a Sterivex-GP Pressure Filter Unit (Merck) immediately after sampling and stored in a frozen state until laboratory processing. Prokaryotic DNA for the molecular bio­logical ana­lysis was extracted using DNeasy PowerWater‍ ‍Sterivex Kits (Qiagen GmbH). Microbes were mechanically disrupted for 10‍ ‍min using ShakeMaster NEO (BioMedical Science), and extracted DNA was stored at –80°C. The abundance of total prokaryotic and archaeal 16S rRNA genes was measured using quantitative real-time polymerase chain reactions (qPCR) with universal and archaea-specific primer-probe sets, respectively (Table S1). The reaction mixture including an innuMIX qPCR MasterMix probe was incubated in a qTOWER^3^ G touch real-time PCR system (Analytik Jena GmbH). Amplification conditions were 50 cycles of denaturation at 98°C for 10‍ ‍s, annealing at 50°C (universal 16S rRNA gene) or 52°C (archaeal 16S rRNA gene) for 45‍ ‍s, and extension at 72°C for 30 s. Calibration curves were constructed using the genomic DNA of *Escherichia coli* and *Methanosarcina barkeri*. All qPCR assays were performed in triplicate.

### Microbial community composition ana­lysis

The hypervariable V3–V4 region of the 16S rRNA gene was amplified by PCR using the universal primers, 341F: 5′-CCTACGGGNGGCWGCAG-3′ and 805R: 5′-GACTACHVGGGTATCTAATCC-3′ (Table S1) (Klindworth *et al.*, 2013). DNA was amplified by PCR using MightyAmp DNA Polymerase Ver.3 (Takara Bio) and a Biometra TAdvanced 96 SG thermal cycler (Biometra). The thermal cycle protocol comprised initial denaturation at 98°C for 5‍ ‍min, then 35 cycles of denaturation at 98°C for 30‍ ‍s, annealing at 55°C for 30‍ ‍s, extension at 68°C for 30‍ ‍s, and a final extension at 68°C for 5‍ ‍min. Potential experimental contamination was assessed by PCR amplification of the negative control for DNA extraction. No PCR amplicons were obtained from the negative control. Amplicons were purified using AMPure XP magnetic beads (Beckman Coulter), indexed using Nextera XT Index Kits (Illumina), and loaded onto the Illumina MiSeq platform for paired-end sequencing. Sequence reads were processed using QIIME 2 2020.8 (Bolyen *et al.*, 2019). Raw sequence data were demultiplexed and quality controlled using DADA2 (Callahan *et al.*, 2016). 16S rRNA gene amplicon sequences were aligned with mafft (Katoh *et al.*, 2002) and used to construct a phylogeny with FastTree (Price *et al.*, 2010). Taxons of representative sequences were identified using a pre-trained naive Bayes classifier (Bokulich *et al.*, 2018) and the q2-feature-classifier plugin. This classifier was trained on Greengenes 13_8 99% OTU full-length sequences (McDonald *et al.*, 2012). Raw sequence data were deposited in the Sequence Read Archive (SRA) under the accession number DRA012339.

### Archaeal 16S rRNA gene cloning, sequencing, and phylogenetic analyses

Archaeal 16S rRNA gene fragments were amplified by PCR using the specific primers listed in Table S1. Among them, the ARCH46f (Øvreås *et al.*, 1997) and 805R (Klindworth *et al.*, 2013) pair amplified ~700 bp of the archaeal 16S rRNA genes under the following conditions: 40 cycles of denaturation at 94°C for 40‍ ‍s, annealing at 52°C for 30‍ ‍s, and extension at 68°C for 60 s. Amplified PCR products were gel-purified, cloned, and subjected to Sanger sequencing as previously described (Yanagawa *et al.*, 2019). Sequences were grouped based on 97% similarity with CodonCode Aligner 6.0.2 (CodonCode). Sequences were checked for chimeras using DECIPHER v.2.8.1 (Wright *et al.*, 2012). Representative sequences were compared with the NCBI database using BLAST searches (Altschul *et al.*, 1990) and aligned with other known 16S rRNA sequences using the SINA Aligner (Pruesse *et al.*, 2012). Taxonomic affiliations were considered based on the SILVA 138 SSURef NR99 database and phylogenetic trees constructed using the neighbor-joining and maximum-likelihood methods in the ARB software package (Ludwig *et al.*, 2004). Confidence values were inferred from phylogenetic trees by bootstrapping 1,000 replicates. The secondary structure of 16S rRNA was generated using R2DT based on a library of reference sequences and the template covariance model (Sweeney *et al.*, 2021). The 16S rRNA gene sequences obtained herein were deposited in the DDBJ/EMBL/GenBank databases under the accession numbers LC640320 and LC640321.

## Results

### Physical and chemical characteristics and abundance of microbes

The temperature range of water from the hot springs was 78.6–95.3°C, and the pH range at the sampling point was slightly alkaline at 7.6–9.2 (Table 1). The chemical composition of hot spring waters was characterized as the neutral NaCl type for both sites. Despite near-boiling temperature conditions close to the limit for microbial growth, cell density ranged between 1.0×10^4^ and 5.9×10^5^‍ ‍cells‍ ‍mL^–1^ of hot water (Table 2). Cells from all sites were morphologically diverse, suggesting various active microbial components (Fig. S2). qPCR results using universal primer/probe sets revealed 3.5×10^4^–4.0×10^5^ whole prokaryotic 16S rRNA genes mL^–1^ of hot water (Table 2). The abundance of archaeal 16S rRNA genes was below the limit of detection (<1.0×10^3^ genes mL^–1^) in most samples.

### 16S rRNA gene-based microbial community structure

**Fig. 2.**
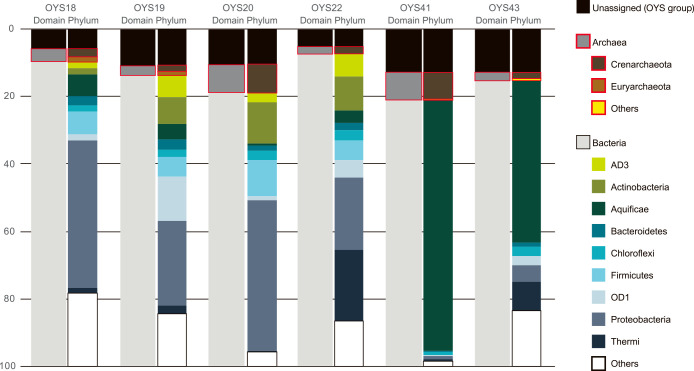
Taxonomic composition of 16S rRNA gene sequences from hot spring water samples around Oyasukyo Gorge. 16S rRNA gene fragments were obtained by HTS.

The composition of the prokaryotic community of the six hot spring samples was assessed by HTS of the V3–V4 hypervariable region of 16S rRNA gene fragments that comprised 104,588 quality-filtered sequences (Table S2). These sequences constituted 740 features, which were created by grouping unique sequences in QIIME 2 (Bolyen *et al.*, 2019). Classification analyses showed that 79.0–92.6% and 2.1–8.5% of the total reads represented bacterial and archaeal 16S rRNA gene sequences, respectively (Fig. 2). Quality-filtered reads consisted of 41 phyla. The dominant phylotypes in the four samples collected in 2016 (OYS18, 19, 20, and 22) belonged to the phylum *Proteobacteria* (21.5–44.7% of the total reads), followed by *Firmicutes* (5.8–10.8%), *Actinobacteria* (1.9–12.3%), and OD1 (1.2–13.2%). *Proteobacteria* members were characterized by the alphaproteobacterial genera *Phyllobacterium* and *Sphingomonas* (1.5–44.7% and 1.6–15.1% of *Proteobacteria* sequences, respectively). None of the members were considered to be original members living in the high-temperature springs. The detected *Firmicutes* involved unclassified members of *Veillonellaceae*, *Geobacillus*, and *Thermoanaerobacterium* (8.1–34.5%, 3.9–56.8%, and 6.0–16.6% of the *Firmicutes* sequences, respectively). *Actinobacteria* sequences mostly belonged to unclassified members of *Acidimicrobiales* (35.2–54.0% of the *Actinobacteria* sequences). In contrast, members of the bacterial phylum *Aquificae*, which thrives in marine and terrestrial hydrothermal environments (Gupta, 2014), were dominant at the OYS41 and 43 sites examined in 2019. They comprised 74.5 and 47.8% of the total reads at the OYS41 and 43 sites, respectively. Most of the members were represented not by any known genera, but by the family *Aquificaceae* (>98.7% of the *Aquificae* sequences). Members of the phylum *Thermi* were also predominant at the OYS22 and OYS43 sites, and most were represented by the genus *Thermus* (82.1–91.1% of the *Thermi* sequences). The organisms belonging to this genus have also been detected in marine and terrestrial hydrothermal systems (Albuquerque and da Costa, 2014). The most numerous archaeal taxa included the class *Thermoprotei* of the phylum *Crenarchaeota*, which comprised <8.1% of the total reads. Most of the members were represented by the genus *Pyrobaculum*. We also detected archaeal phylotypes of the classes *Aigarchaeota* (<1.9% of the total reads), *Archaeoglobi* (<1.4%), and *Thermoplasmata* (<1.0%).

Notably, 10,416 sequences of 385 bp did not fall into known clades at the domain level. All of the unassigned sequences were classified as archaea by the naïve Bayesian classification method, the RDP Classifier (Wang *et al.*, 2007), and formed an independent clade (Fig. S3). This indicated that they were derived from the 16S rRNA genes of a new deep-branching phylogenetic lineage of archaea. These sequences, represented by the OYS group, were shared among all of the samples analyzed and comprised 5.2–12.9% of the microbial community (Fig. 2).

### Phylogenetic analyses of the OYS group using longer sequences based on cloning

**Fig. 3.**
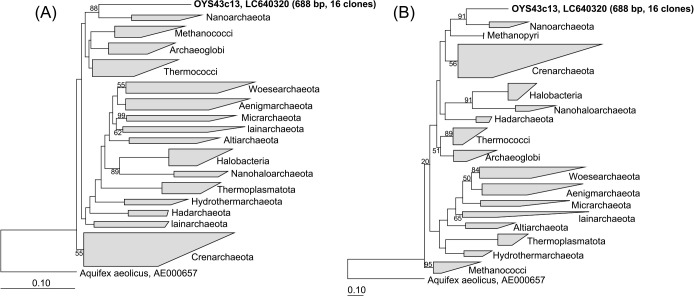
Phylogenetic tree of archaeal 16S rRNA gene sequences based on neighbor-joining (A) and maximum-likelihood (B) methods. Sequences obtained by a clone library ana­lysis are indicated in bold. The sequence length and number of OTUs are shown in parentheses. Values at nodes represent bootstrap scores >50%. Bootstrap values are expressed as ratios (%) of 1,000 replicates. Scale bar, 10% estimated sequence divergence.

Full-length 16S rRNA gene sequences were ideal for a more detailed phylogenetic ana­lysis; however, OYS group sequences were not obtained in clone libraries constructed using various appropriate primers (Table S1). Only the ARCH46f-805R primer pair amplified 688 bp of OYS sequences from the OYS43 site. These sequences accounted for 57.1% of the clone library (Fig. S4). A similarity ana­lysis of the cloned sequence OYS43c13 revealed only distant relationships with all of the other 16S rRNA genes examined, and very low identities with environmental clone sequences in the NCBI database (Table 3). The top BLAST hit was the uncultured *Desulfurococcaceae* clone found in a deep-sea hydrothermal vent (accession number AB095128, 81.61% identity). The BLAST search set to exclude environmental clone sequences also indicated a close relationship with *Desulfurococcaceae* isolates (Table S3). The phylogenetic tree ana­lysis using the neighbor-joining method showed the distinct separation of the OYS group from the phylum *Nanoarchaeota*, with a bootstrap value of 88% (Fig. 3A). This was consistent with the unrooted maximum likelihood phylogenetic tree shown in Fig. 3B, which also showed that the OYS group was distinctly separate from *Nanoarchaeota*, with a bootstrap value of 91%. This result suggested that the OYS group has no known close relatives and is a deeply branching novel archaeal lineage. In spite of its unique phylogenetic position, the secondary structure of OYS 16S rRNA showed rRNA-like stems and loops (Fig. S5).

## Discussion

### Phylogenetic position of the OYS phylotype

OYS group archaea were distantly related to known phyla. Phylogenetic analyses of 16S rRNA gene amplicons with relatively short sequences revealed that the OYS group clustered together as a basal branch of the phyla *Crenarchaeota*, *Korarchaeota*, *Heimdalarchaeia*, and *Odinarchaeia* (Fig. S3). However, the relatively short 16S rRNA gene fragments obtained through the microbial community composition ana­lysis were generally insufficient for accurately locating the phylogenetic position (Johnson *et al.*, 2019). Cloning provided more reliable information on OYS 16S rRNA gene sequences, which were ~1.8-fold longer than those obtained from the community ana­lysis. The naïve Bayesian classification method and phylogenetic ana­lyses using neighbor-joining and maximum likelihood trees showed the robust placement of the OYS group as a new phylum-level archaeal lineage that was distantly related to all known phylogenetic clades (Fig. 3). The OYS group was relatively close to *Nanoarchaeota*, and showed a similarity of 76.93% to the cultured representative, *Nanoarchaeum equitans* (AJ318041). However, the deep branch isolating the OYS group from *Nanoarchaeota* and the high bootstrap value of the node indicated that they were distinctly separate from each other. This is consistent with the result showing that the “closest” relative belongs to the *Desulfurococcaceae* clone with 81.61% similarity (Table 3). Even if *Nanoarchaeota* is a sister group of the OYS archaea, we were unable to accurately resolve its phylogenetic position. The exact branching point of *Nanoarchaeota* within the archaeal phylogenetic tree remains obscure because its phylogenetic position may vary according to the algorithms and parameters applied (Brochier *et al.*, 2005). Further phylogenetic analyses of concatenated protein sequences from the OYS group will provide novel insights into its phylogenetic relationship with other distinct lineages.

### Overlooked OYS group

OYS group archaea may have been overlooked largely due to the low sequence coverage and low amplification efficiency of popular archaeal or prokaryotic primers (Bahram *et al.*, 2019). Only ARCH46f/805R among the 18 archaea-specific or universal primers (Table S1) amplified the OYS sequences by PCR, suggesting that these primers have less coverage of the OYS sequences. All OYS sequences had at least one mismatch with the forward primer, which is conventionally used for cloning or qPCR. A109f, which is often used to amplify archaeal 16S rRNA genes, had four mismatches with the OYS sequence. A similar discussion of qPCR results may be relevant because they mostly showed that the number of archaeal 16S rRNA genes was below the detection limit. Since the OYS sequences were detected using 341F/805R and ARCH46f/805R, the archaea-specific primer-probe set for qPCR may not amplify the OYS group. The forward primer Arch349F and the TaqMan probe Arch516F had one and two mismatches with the OYS sequence, respectively. Therefore, the unique 16S rRNA gene in the OYS group may show some variations in the universally conserved primer targets, which may result in difficulties with its detection, even with popular primers.

### Are OYS archaea thermophilic?

The near-boiling temperature of the OYS site, which reaches 95.3°C, indicates a unique thermal environment because water at atmospheric pressure boils at ~100°C. The hot spring temperature at the OYS site is within the growth temperature range of hyperthermophiles. The microbial biomass reaches 5.9×10^5^‍ ‍cells‍ ‍mL^–1^ even at extremely high temperatures. Abundance is similar in other terrestrial hot springs with high temperatures (>76°C). These community members are characterized by organisms closely related to *Desulfurococcales* and *Aquificae* in Philippines hot springs (Huang *et al.*, 2013), *Aquificae* and *Thermoprotei* in Bourlyashchy Pool, Kamchatka, Russia (Chernyh *et al.*, 2015), *Desulfurococcales* and unclassified *Crenarchaeota* in Yunnan and Tibetan hot springs (Song *et al.*, 2013), *Thermocrinis* (*Aquificae*) in Great Boiling Spring, US Great Basin (Cole *et al.*, 2013), *Firmicutes*, *Proteobacteria*, and *Hydrogenobacter* (*Aquificae*) in Malaysian hot springs (Chan *et al.*, 2015), and *Proteobacteria* and *Chloroflexi* in the Himalayan geothermal region (Amin *et al.*, 2017). *Nanoarchaeota* also has a widespread distribution in terrestrial hot spring environments with similar temperature conditions (Clingenpeel *et al.*, 2013). On the other hand, this study found molecular biological evidence of a hitherto undiscovered archaeal group thriving in boiling hot springs in Japan. The OYS group represented the most abundant archaeal components in the microbial communities at the study sites. These components were shared among all of the samples obtained in 2016 and 2019, indicating that they are indigenous in the high-temperature fluids around Oyasukyo Gorge. The hot springs harbor prominent members of *Aquificaceae* and *Pyrobaculum* as well as the OYS group, some of which are extreme hyperthermophiles that grow at temperatures exceeding 85°C (Huber *et al.*, 1992). Therefore, we considered the OYS group to have hyperthermophilic properties. On the other hand, fluctuations in high-temperature hydrothermal fluids constrain habitability for microbes (Yanagawa *et al.*, 2017). Spatiotemporally variable conditions may provide a gap between *in situ* and laboratory temperatures for the optimal growth of microorganisms. Therefore, the possibility that the OYS group are thermophiles cannot be denied. 16S rRNA sequences closely related to thermophiles, such as *Thermus* (Albuquerque and da Costa, 2014) and *Thermoplasmata* (Reysenbach and Brileya, 2014), were concomitantly detected in the same samples.

The prediction of physiological characteristics using only rRNA gene sequence data is generally impossible. Nevertheless, the GC contents of 16S rRNA gene sequences closely correlated with the growth temperature ranges of archaea (Kimura *et al.*, 2010). This study enabled estimations of the minimum, optimal, and maximum growth temperatures of uncultured archaea. The GC content of the OYS 16S rRNA gene sequence OYS43c13 was 64.7%: however, the sequence obtained using ARCH46f and 805R did not cover the entire region used for molecular thermometry. Assuming that this value was consistent in the entire 16S rRNA gene, we estimated the potential minimum, optimal, and maximum growth temperatures of OYS to be 58.2, 81.3, and 88.4°C, respectively. These estimated values were consistent with the temperatures measured at the sampling sites. Therefore, OYS group archaea may represent an active population of discharged water.

### Locations of OYS growth

We discovered a previously unknown lineage of archaea in boiling hot springs. However, their actual and active habitats remain enigmatic. We herein suggest that their habitat is not limited to the vicinity of the spring orifice, but extends to the shallow subsurface beneath the studied site. High microbial abundance in a significant amount of discharged water at Oyasukyo Daifunto may be derived from the underground biosphere with a temperature exceeding the boiling point of water at atmospheric pressure. On the other hand, the results of chemical geothermometry based on silica concentrations assuming quartz saturation (Fournier, 1977) indicated that the reservoir temperature of the hot spring waters studied was at least 150°C. Moreover, chemical geothermometry based on the alkali element composition (Fournier and Truesdell, 1973) implied a markedly higher temperature. Therefore, the underground hot water reservoir appears to be an unlikely source of the OYS group because the upper temperature limit for the growth of culturable hyperthermophiles is 122°C, and higher temperatures decrease viability within a few hours (Takai *et al.*, 2008). OYS archaea appear to comprise an indigenous population in the subsurface environment if it is shallower than the reservoir and has a relatively mild temperature close to the upper temperature limits of life.

## Conclusions

The discovery of a novel hyperthermophilic lineage expands our understanding of microbial physiology in high-temperature extremes and further applications to biotechnology and bioremediation. The present study showed that even historically investigated environmental habitats, such as hot springs, possess unanticipated 16S rRNA genes representing a novel phylum-level archaeal lineage. We applied a fairly simple and classical approach using PCR to discover abundant novel species in the OYS group from high-temperature hot springs. These species may have remained overlooked because of their unique sequences. Their novelty may be ascribed to the distinct geochemical and/or physical properties of the hot springs. The underlying environmental factors that elevated the relative abundance of OYS group archaea were not clarified in the present study. Genome sequencing of this deeply rooted microorganism will be a high-priority project to understand its physiological functions and establish a more resolved phylogenetic placement in the archaeal tree. The present results will facilitate the isolation of members of the OYS lineage. Japan has numerous hot springs with unique geochemical characteristics and geological backgrounds, as well as microbial communities that await investigation. Further studies on these important natural analogs of early Earth will provide insights into the evolution and origin of life and habitability on other planets.

## Citation

Asamatsu, K., Yoshitake, K., Saito, M., Prasitwuttisak, W., Ishibashi, J., Tsutsumi, A., et al. (2021) A Novel Archaeal Lineage in Boiling Hot Springs around Oyasukyo Gorge (Akita, Japan). *Microbes Environ ***36**: ME21048.

https://doi.org/10.1264/jsme2.ME21048

## Supplementary Material

Supplementary Material

## Figures and Tables

**Table 1.** Geochemical characteristics of hot water samples.

Sample ID	Site	Sampling date	Temp. °C	pH	EC mS m^–1^	ORP mV	SiO_2_ mM	NH_3_ μM	H_2_S μM	Alkalinity meq L^–1^	Na mM	Mg μM	K μM	Ca μM	Fe μM	Al μM	Cl mM	SO_4_ mM
OYS18	Oyasukyo Daifunto	2016/09/27	95.3	7.6	121	–267	2.78	606	19	1.00	7.04	<2	473	596	<0.90	<1.9	4.19	1.28
OYS19	Oyasukyo Daifunto	2016/09/27	93.2	8.2	143	–273	3.23	606	23	1.11	8.44	<2	553	589	<0.90	<1.9	4.70	1.39
OYS20	Oku-Oyasukyo Ooyu	2016/09/27	93.1	8.7	134	–371	3.75	652	51	1.55	9.35	<2	490	189	<0.90	<1.9	4.25	1.18
OYS22	Storage tank	2016/09/27	78.6	9.2	100	106	2.29	560	0.5	0.67	6.38	<2	423	408	<0.90	<1.9	4.01	1.11
OYS41	Oku-Oyasukyo Ooyu	2019/11/15	90.4	9.2	—	—	—	—	—	—	—	—	—	—	—	—	—	—
OYS43	Oyasukyo Daifunto	2019/11/16	90.4	8.4	—	—	—	—	—	—	—	—	—	—	—	—	—	—

**Table 2.** Microbial cell counts and qPCR findings of 16S rRNA gene abundance.

Sample ID	Total counts (cells mL^–1^)	Prokaryotic 16S rRNA (genes mL^–1^)	Archaeal 16S rRNA (genes mL^–1^)
OYS18	3.1±0.33×10^4^	5.5±0.66×10^4^	<1.0×10^3^
OYS19	2.3±0.28×10^4^	4.3±0.92×10^4^	<1.0×10^3^
OYS20	2.6±0.90×10^4^	3.6±0.22×10^4^	<1.0×10^3^
OYS22	5.9±0.98×10^5^	4.0±2.00×10^5^	2.4±1.0×10^3^
OYS41	1.0±0.69×10^4^	3.5±0.33×10^4^	<1.0×10^3^
OYS43	1.9±0.14×10^4^	5.4±0.57×10^4^	<1.0×10^3^

Data are shown as means±standard deviations.

**Table 3.** BLAST search result of OYS 16S rRNA sequence obtained from the OYS43 cloning library.

OTU	Length (bp)	Top hit (accession No.)	Taxonomic affiliation*	Identity	Query cover	E	Reference
OYS43c13	688	pCIRA-S (AB095128)	Archaea; *Crenarchaeota*; *Thermoprotei*; *Desulfurococcales*; *Desulfurococcaceae*	81.61% (546 out of 669)	96%	6E-149	Takai *et al.*, 2004

* Elucidated using RDP Hierarchy Browser.
