# Supplementary material for: A Novel Archaeal Lineage in Boiling Hot Springs around Oyasukyo Gorge (Akita, Japan)
Source: Microbes Environ. 2021 Nov 25;36(4):ME21048. doi: 10.1264/jsme2.ME21048 (PMC8674440; doi:10.1264/jsme2.ME21048)
Supplement: Supplementary file 1 — Supplementary Material [file 36_21048_s1.pdf]

Supplemental Material

A novel archaeal lineage in boiling hot springs around Oyasukyo Gorge (Akita, Japan)

Katsuhiro Asamatsu, Kai Yoshitake, Makoto Saito, Wipoo Prasitwuttisak, Jun-ichiro Ishibashi, Akihi Tsutsumi, Nurul Asyifah Mustapha, Toshinari Maeda, and Katsunori Yanagawa

Table S1. Primers and probes for PCR and qPCR.

Primer/Probe	Target 16S rRNA genes	Procedure	Sequence (5'-3')	Target sequence region of OYS group (5'-3')	Mismatch
341F	Prokaryotic	NGS	CCTACGGGNGGCWGCAG	CCTACGGGTCGCAGCAG	1
805R			GACTACHVGGGTATCTAATCC	-	
Uni340F	Prokaryotic	qPCR	CCTACGGGRBGCASCAG	CCTACGGGTCGCAGCAG	1
Uni806R			GGACTACNNGGGTATCTAAT	-	
Uni516F (Taqman probe)			TGYCAGCMGCCGCGGTAAHACVNRS	TGGCAGCCGCCACGGTAATACCGGC	2
Arch349F	Archaeal	qPCR	GYGCASCAGKCGMGAAW	TCGCAGCAGGCGCGAAA	1
Arch806R			GGACTACVSGGGTATCTAAT	-	
Arch516F (Taqman probe)			TGYCAGCCGCCGCGGTAAHACCVGC	TGGCAGCCGCCACGGTAATACCGGC	2
Arc9F	Archaeal	Cloning	CYGGTYGATCCYGCCRG	-	
Arc21F	Archaeal	Cloning	TTCCGGTTGATCCYGCCGG	-	
ARCH46f	Archaeal	Cloning	YTA AGC CAT GCR AGT	-	
A109f	Archaeal	Cloning	AHDGCTCAGTAACACRT	ACTGCTGAGTAATACCA	4
Arc958R	Archaeal	Cloning	YCCGGCGTTGAMTCCAATT	-	
Arch 907R	Archaeal	Cloning	CCGCCAATTCCTTTAAGTTT	-	
ARCH915R	Archaeal	Cloning	GTGCTCCCCCGCCAATTCCT	-	
A932R	Archaeal	Cloning	GCYCYCCCGCCAATTCMTTTA	-	
Arch958R	Archaeal	Cloning	YCCGGCGTTGAMTCCAATT	-	
SSU1000ArR	Archaeal	Cloning	GGCCATGCAMYWCCTCTC	-	
Arch1017R	Archaeal	Cloning	GGCCATGCACCWCCTCTC	-	
1114aR	Archaeal	Cloning	GGGTCTCGCTCGTTRCC	-	
A1206R	Archaeal	Cloning	TTGYAGCCGCGTGHGCCC	-	
A1335R	Archaeal	Cloning	GTGTGCAAGGAGCAGGGAC	-	
A1389R	Archaeal	Cloning	ACGGGCGGTGTGTGCAAG	-	
U1392R	Prokaryotic	Cloning	ACGGGCGGTGTGTRC	-	
Univ1490R	Prokaryotic	Cloning	GGHTACCTTGTTACGACTT	-	
Univ1492R	Prokaryotic	Cloning	GGTTACCTTGTTACGACTT	-	

NGS, next-generation sequencing; qPCR, quantitative polymerase chain reaction.

Table S2. Overview of 16S rRNA gene amplicon sequencing.

Sample ID	number of quality-filtered reads	number of features
OYS18	16951	153
OYS19	14301	166
OYS20	14846	70
OYS22	15940	88
OYS41	20685	100
OYS43	21865	303

Table S3. Top 5 sequences of the BLAST search set to exclude uncultured/environmental sample sequences.

OTU	Accession	Taxonomy	Identity	Query cover	E
OYS43c13	NR_029316	Archaea; Crenarchaeota; Thermoprotei; Desulfurococcales; Desulfurococcaceae; Sulfophobococcus	81.14%	96%	2E-144
	NR_133017	Archaea; Crenarchaeota; Thermoprotei; Desulfurococcales; Desulfurococcaceae; Thermogladius	80.84%	96%	2E-140
	EF088324	Archaea; Crenarchaeota; Thermoprotei; Desulfurococcales; Desulfurococcaceae; Desulfurococcus; unclassified Desulfurococcus	80.69%	95%	9E-138
	Y07963	Archaea; Crenarchaeota; Thermoprotei; Desulfurococcales; Desulfurococcaceae; Staphylothermus	80.68%	96%	9E-138
	NR_148751	Archaea; Crenarchaeota; Thermoprotei; Desulfurococcales; Desulfurococcaceae; Thermogladius	80.39%	96%	2E-135

Supplementary figures

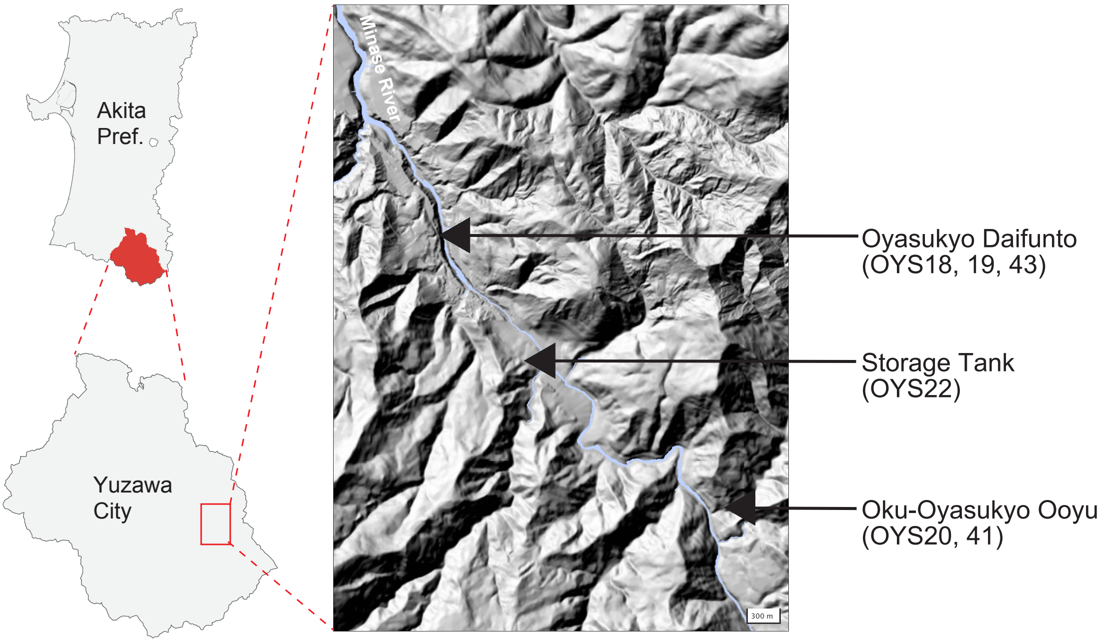

Fig. S1. Detailed map of the research area. The shaded relief map was provided by the Geospatial Information Authority of Japan.

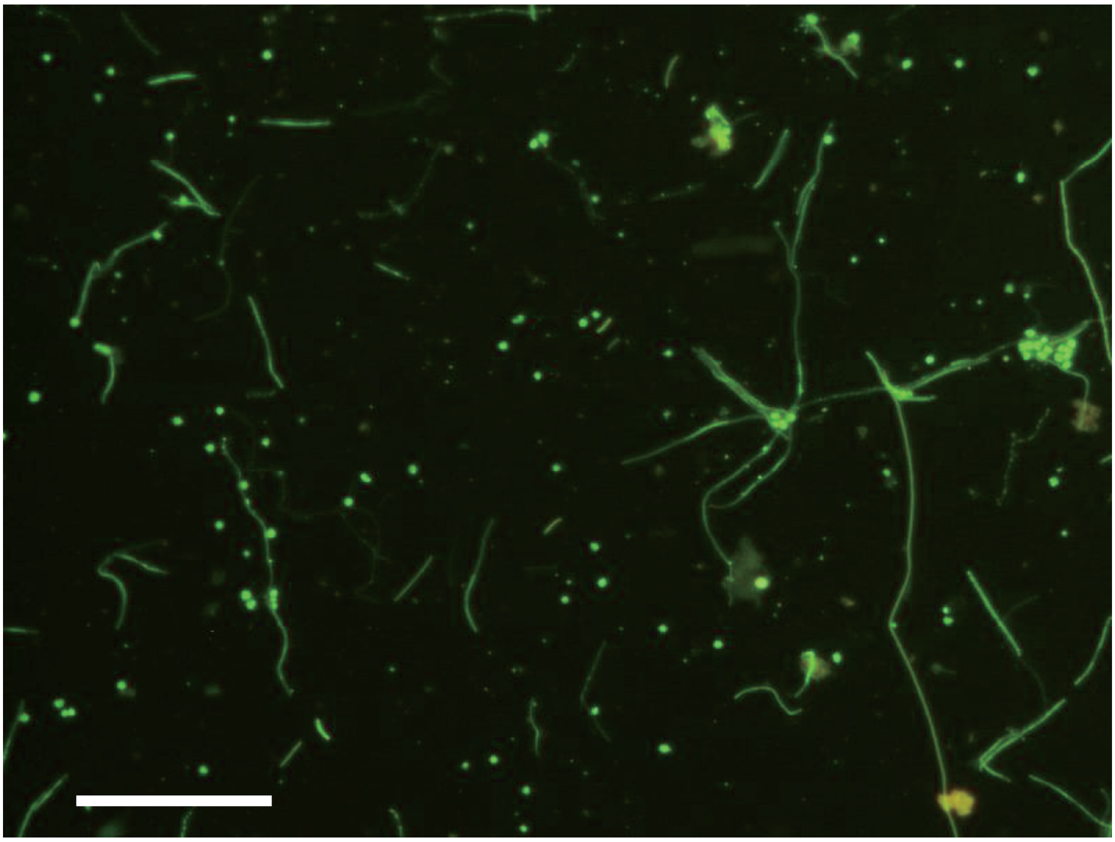

Fig. S2. Fluorescence microscopy image of microbial cells from OYS19 site, which is selected as an example to show cell morphology. Cells are stained with SYBR Green I. Bar indicates 10 μm.

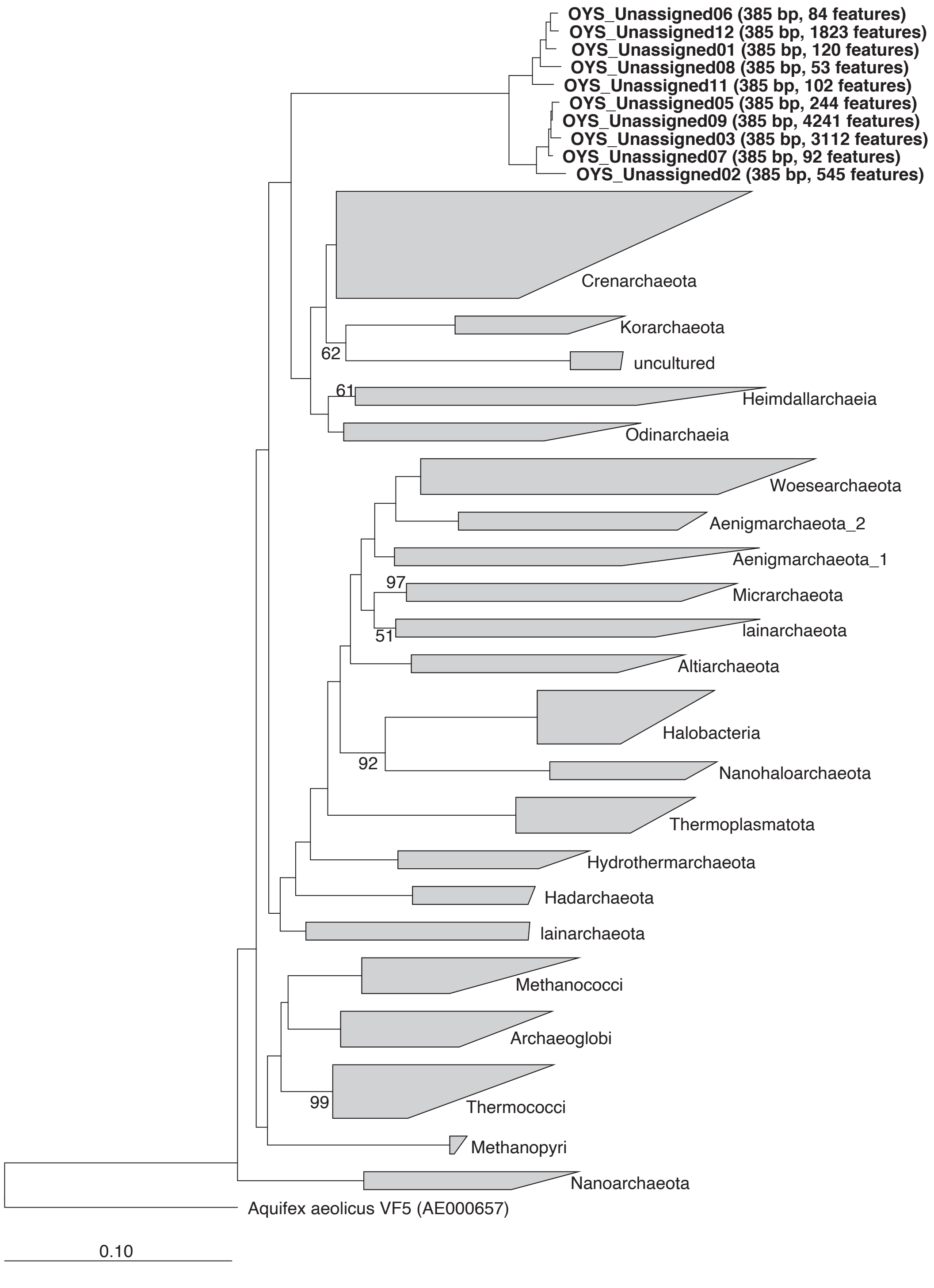

Fig. S3. Neighbor-joining phylogenetic tree of representative sequences obtained by HTS of 16S rRNA gene amplicons. The boldface type indicates the sequences obtained in this study. The sequence length and the number of sequence features are shown in parentheses. Values at nodes represent bootstrap scores > 50%. Bootstrap values are expressed as ratios (%) of 1,000 replicates. Scale bar, 10% estimated sequence divergence.

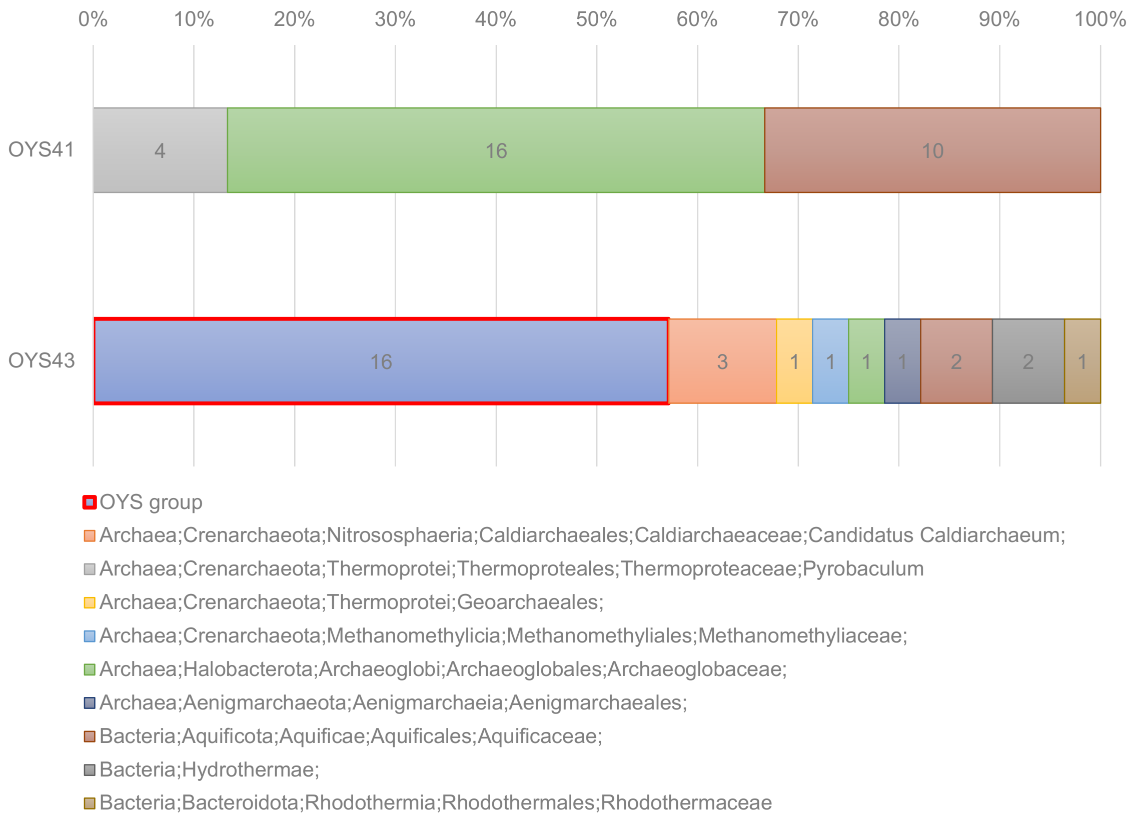

Fig. S4. Microbial community structures of the OYS41 and 43 clone libraries constructed using the ARCH46f-805R primer pair. The number of clones is indicated within each bar.

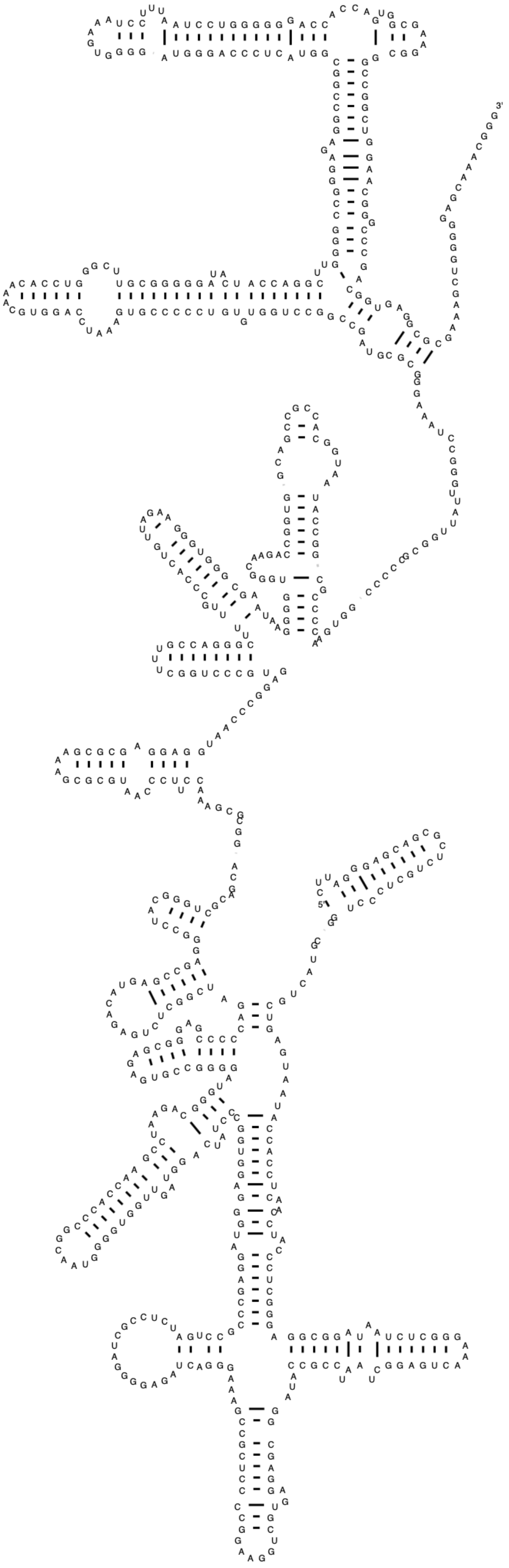

Fig. S5. Secondary structure of the partial 16S rRNA of the OYS group clone OYS43c13. The model structure was determined using R2DT.
